# Small-Cell Carcinoma of the Gall Bladder: A Rare Tumor of the Gall Bladder

**DOI:** 10.7759/cureus.53172

**Published:** 2024-01-29

**Authors:** Anuradha S Dnyanmote, Kuldip Patil, Vidita Modi, Rushi Kanani

**Affiliations:** 1 Surgery, Dr. D. Y. Patil Medical College, Hospital and Research Centre, Pune, IND; 2 General Surgery, Dr. D. Y. Patil Medical College, Hospital and Research Centre, Pune, IND; 3 General Surgery, Dr. D.Y. Patil Vidyapeeth, Pune, IND

**Keywords:** small-cell carcinoma, resection, cholecystectomy, chemotherapy, neuroendocrine tumours

## Abstract

Small-cell carcinoma (SCC) of the gallbladder is a rare and distinctive clinicopathological entity, characterized by its aggressive nature with early metastasis and a poor prognosis. We present a rare case of a 53-year-old female who came with a perforated gall bladder and was later diagnosed with SCC. This report details how the patient was managed preoperatively, intraoperatively, and postoperatively. The patient is under follow-up and has survived so far with subsequent chemotherapy.

## Introduction

First introduced by Albores-Saavedra et al. in 1981, small-cell carcinoma (SCC) of the gallbladder remains exceptionally uncommon [1]. There is still a lack of comprehensive data on this malignancy, with only 73 cases documented in the English literature as of now. Given the advanced stage at presentation and the highly malignant nature of the tumor, the prognosis for SCC of the gallbladder is notably poor, with a reported median survival time of nine months [2]. The optimal treatment approach is not well-defined, but patients with localized disease in the gallbladder may undergo a combination of therapeutic interventions, including surgical resection and chemoradiation [3].

## Case presentation

A 53-year-old female presented to the emergency department with chief complaints of pain in the abdomen and fever for three days. The patient also has a history of cholelithiasis for which no intervention was done. The patient was vitally stable, and upon physical examination, tenderness was present over the epigastric, right hypochondrium, and right lumbar region. Except for leukocytosis, all labs were within normal limits. USG Abdomen pelvis and CECT Abdomen pelvis were both suggestive of a thickened gall bladder with suspicious gall bladder perforation with necrotic lymph nodes in the peri-pancreatic region.

**Figure 1:**
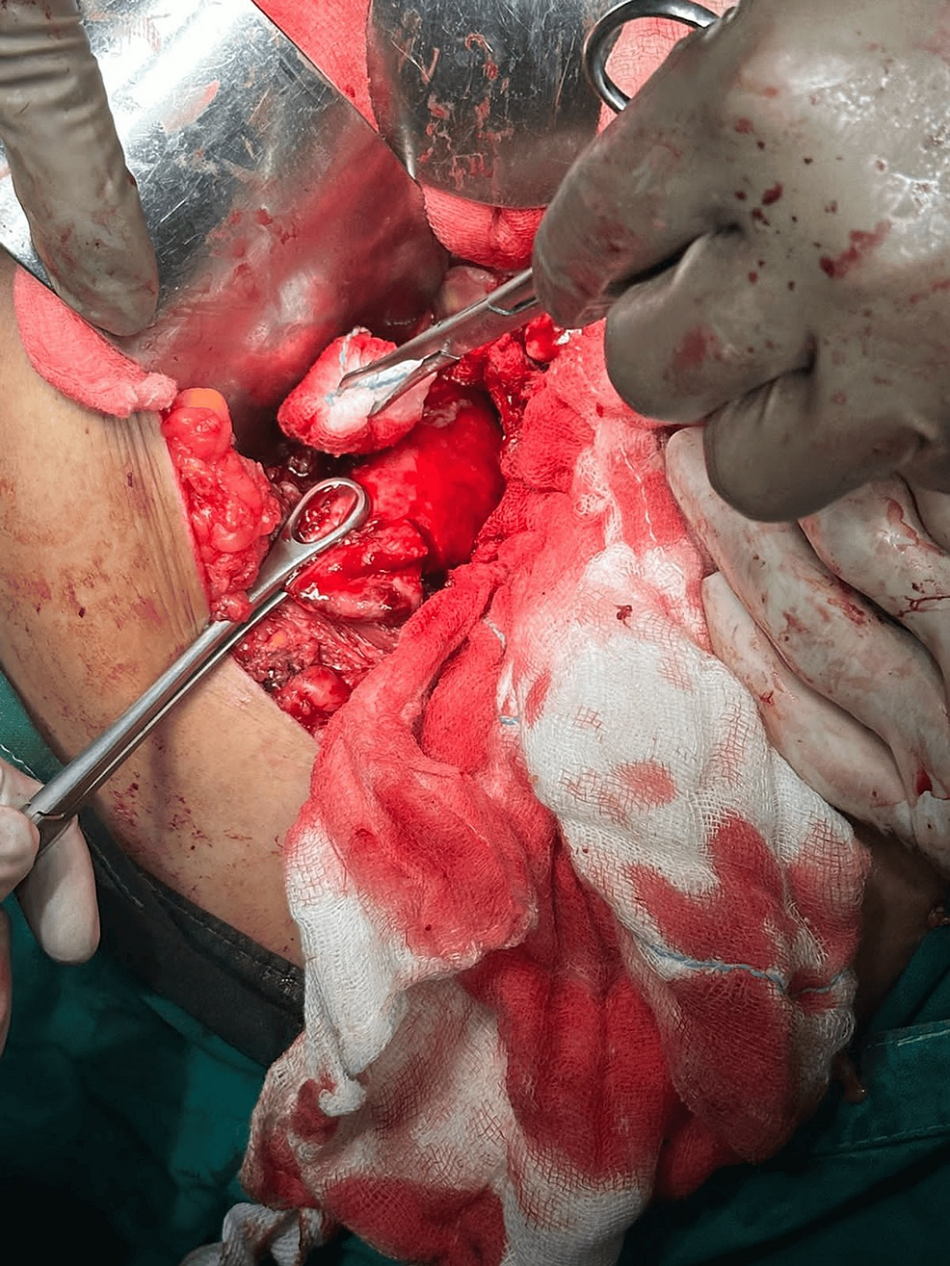
Intraoperative perforated gall bladder

**Figure 2:**
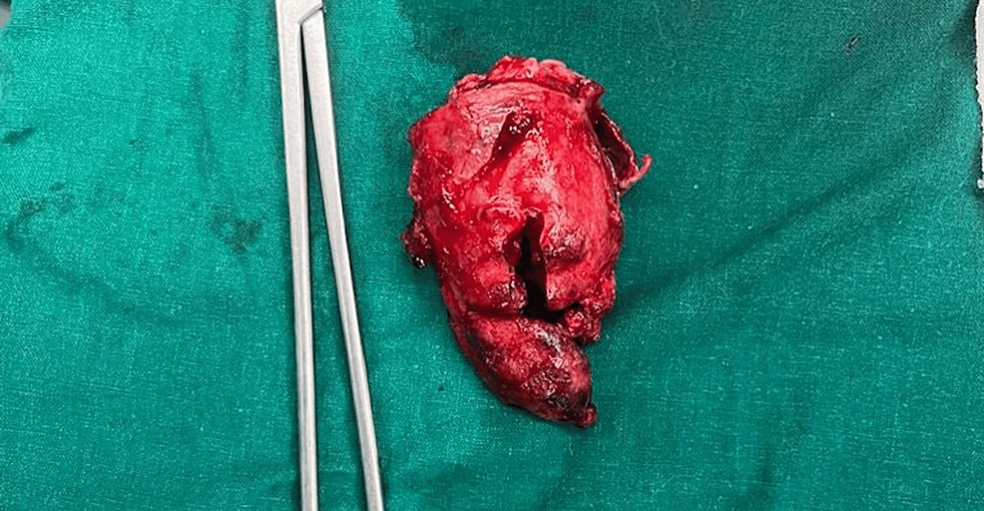
Specimen of excised gall bladder

The patient was explained about the condition and with proper consent, the patient was taken up for surgery. The patient underwent exploratory laparotomy, revealing a perforated gall bladder intraoperatively (Figure 1), for which a total cholecystectomy was performed (Figure 2).

Histopathology findings were suggestive of SCC - gall bladder, and IHC markers (Synaptophysin, CD56, Ki67, EMA, CK19) sent for confirmatory findings were also raised. Post-op PET CT showed periportal, paracaval, and aortocaval metastatic lymph nodes. The chemotherapy regimen (cisplatin + etoposide) was started for the patient postoperatively, and the patient has been under follow-up. The patient has survived so far with subsequent chemotherapy.

## Discussion

Gallbladder carcinoma ranks as the fifth most prevalent gastrointestinal malignancy, witnessing around 7,000 new diagnoses annually in the United States. The majority of these cases are characterized by adenocarcinoma [4,5]. Few well-established risk factors for this disease include choledochal cysts, gallstones larger than three centimeters, and prolonged inflammatory conditions. Notably, due to the absence of systemic symptoms, gallbladder carcinoma frequently presents in advanced stages, with merely ten percent of cases restricted to the gallbladder wall upon diagnosis [6]. This subtle onset contributes to an overall survival rate of less than 5%. Currently, there is limited research dedicated to the medical treatment of this condition, with many trials lacking sufficient power or including diverse tumor types. Adjuvant chemotherapy is commonly employed but lacks a standardized protocol. The available therapeutic options are primarily centered around complete surgical resection.

Neuroendocrine carcinomas represent an even smaller percentage of the general surgeon’s practice. The occurrence of SCC in the gallbladder constitutes about 0.5% of all gallbladder cancers, with adenocarcinoma being the predominant type [7]. In the realm of neuroendocrine tumors within the gastrointestinal tract, SCC of the gallbladder accounts for 0.2% [8]. Typically, it manifests in older women and is frequently associated with the presence of gallstones. Additionally, this ailment is commonly identified in its advanced stages during diagnosis. Approximately two-thirds of individuals receive a diagnosis at stage IV. The median survival duration is nine months. The primary locations for metastasis include the lymph nodes, liver, and lungs. Notably, there is no documentation of SCC of the gallbladder exhibiting carcinoid syndrome or other symptoms induced by biologically active peptides.

Information regarding the management of SCC of the gallbladder is limited. The rarity of this tumor, coupled with its tendency to present in advanced stages due to its aggressive nature, poses challenges in establishing standardized management protocols. While surgery remains the primary approach for treating gallbladder cancer, the effectiveness of radical procedures in improving outcomes for advanced stages of SCC is uncertain. In a study examining hepatopancreatobiliary SCC, Groeschl et al. examined the SEER (Surveillance, Epidemiology, and End-Results) database from 1998 to 2008 and concluded that surgical resection correlated with extended survival in patients with localized pancreatobiliary SCC [3].

Given the unfavorable prognosis associated with SCC of the gallbladder, even in cases where curative surgery is feasible, it is advisable to explore supplementary approaches such as chemoradiation. Similar to its efficacy in pulmonary SCC, the combination of etoposide and cisplatin has demonstrated effectiveness in treating patients with SCC of the gallbladder. As previously documented, gemcitabine can also be combined with platinum agents for administration in these cases [9]. SCC of the gall bladder has an extremely low incidence and poor prognosis. Early detection and resection of the gall bladder along with chemotherapy seem to have prolonged survival and better outcomes.

## Conclusions

The management of SCC of the gallbladder is particularly challenging, given its tendency for late detection and the associated poor prognosis. As illustrated in our case, metastatic lymph nodes in periportal, paracaval, and aortocaval regions were already evident. Due to the scarcity of information on the treatment of this rare condition, the chosen approach involved excision followed by chemotherapy. The patient has been under continuous follow-up since then with subsequent chemotherapy. 

## References

[1] Albores-Saavedra J, Cruz-Ortiz H, Alcantara-Vazques A, Henson DE (1981). Unusual types of gallbladder carcinoma. A report of 16 cases. Arch Pathol Lab Med.

[2] Mahipal A, Gupta S (2011). Small-cell carcinoma of the gallbladder: report of a case and literature review. Gastrointest Cancer Res.

[3] Groeschl RT, Christians KK, Turaga KK, Gamblin TC (2013). Management of primary hepatopancreatobiliary small cell carcinoma. J Surg Oncol.

[4] Pavithran K, Prabhash K, Hazarika D, Doval DC (2010). Neuroendocrine carcinoma of gallbladder: report of 2 cases. Hepatobiliary Pancreat Dis Int.

[5] Lane JE, Walker AN, Ayers GW, Foster JL, Williams JT (2002). Small-cell undifferentiated carcinoma of neuroendocrine type originating in the gallbladder. Curr Surg.

[6] Boerma EJ (1994). Towards an oncological resection of gall bladder cancer. Eur J Surg Oncol.

[7] Henson DE, Albores-Saavedra J, Corle D (1992). Carcinoma of the gallbladder. Histologic types, stage of disease, grade, and survival rates. Cancer.

[8] Modlin IM, Lye KD, Kidd M (2003). A 5-decade analysis of 13,715 carcinoid tumors. Cancer.

[9] Bahadur S, Shaukat A, Gibbs J, Litwin A, Nava H, Melnyk M, Javle M (2005). Cisplatin and gemcitabine for small cell carcinoma of the gall bladder. Am J Clin Oncol.

